# The epidemiology of primary and secondary adrenal malignancies and associated adrenal insufficiency in hospitalised patients: an analysis of hospital admission data, NSW, Australia

**DOI:** 10.1186/s12902-021-00787-6

**Published:** 2021-07-03

**Authors:** Anna Lubomski, Henrik Falhammar, David J. Torpy, R. Louise Rushworth

**Affiliations:** 1 School of Medicine, Sydney, The University of Notre Dame Australia, Darlinghurst, Australia; 2 Department of Endocrinology, Metabolism and Diabetes, Karolinska University Hospital, 141 86 Stockholm, Sweden; 3 Department of Molecular Medicine and Surgery, Karolinska Institutet, 171 76 Stockholm, Sweden; 4 Endocrine and Metabolic Unit, Royal Adelaide Hospital and University of Adelaide, North Terrace, Adelaide, Australia

**Keywords:** Adrenal malignancy, Adrenal insufficiency, Adrenal crisis, Epidemiology

## Abstract

**Background:**

Adrenal insufficiency (AI) causes considerable morbidity but may remain undiagnosed in patients with adrenal malignancy (AM). The epidemiology of AI and adrenal crises (AC) in AM is uncertain.

**Methods:**

This was a retrospective study examining hospital admission data from 2006 to 2017. All admissions to all hospitals in NSW, Australia over this period with a principal or comorbid diagnosis of an adrenal malignancy were selected. Data were examined for trends in admissions for AM and associated AI/AC using population data from the corresponding years.

**Results:**

There were 15,376 hospital admissions with a diagnosis of AM in NSW over the study period, corresponding to 1281 admissions/year. The AM admission rate increased significantly over the study period from 129.9/million to 215.7/million (*p* < 0.01). An AI diagnosis was recorded in 182 (1.2%) admissions, corresponding to an average of 2.1/million/year. This rate increased significantly over the years of the study from 1.2/million in 2006 to 3.4/million in 2017 (*p* < 0.01). An AC was identified in 24 (13.2%) admissions with an AI diagnosis. Four patients (16.7%) with an AC died during the hospitalisation.

**Conclusion:**

Admission with a diagnosis of AM has increased over recent years and has been accompanied by an increase in AI diagnoses. While AI is diagnosed in a small proportion of patients with AM, ACs do occur in affected patients.

## Background

Adrenal tumours affect approximately 3 to 10% of the population and have become more common over recent years, largely due to an increase in imaging investigations for non-adrenal illnesses [1–4]. Most adrenal tumours are small, benign, non-functioning adrenocortical adenomas, which increase in incidence with advancing age [1, 4]. By comparison, malignant tumours of the adrenal gland are less common, accounting for 2.8 to 6.7% of all adrenal neoplasms [3, 5–7] and are most often due to metastatic spread to the adrenals from a primary malignant tumour in another organ, with common primary sites being lung, breast, melanoma and, less frequently, carcinomas of the stomach, colon, rectum, and pancreas [3, 7–9]. In contrast to the incidence of secondary malignancies, primary adrenal malignancies are rare, have an estimated incidence of 7.4/million [1], and are most often adrenocortical carcinomas (ACC), which occur at approximately 1–2 cases per million per year [10]. Malignant pheochromocytomas and primary adrenal lymphomas are rarer forms of primary adrenal tumours [11, 12]. Adrenalectomy is used to treat most primary malignant adrenal tumors and may also be used to treat adrenal metastases, either as part of a wider surgical procedure to remove a primary lesion in another organ, such as a renal tumor, or as a standalone operation.

Adrenal insufficiency (AI) is an important sequela of malignant disease in the adrenal gland that becomes clinically apparent when approximately 90% of the glandular tissue is destroyed [7]. Hypoadrenalism in patients with adrenal malignancies may also occur due to other factors including pituitary metastases; hypophysitis, with pituitary hormone deficits, or more rarely adrenalitis, following immunotherapies for certain malignancies, such as melanoma; and the use of the adrenolytic agent mitotane to treat adrenocortical carcinoma [13, 14]. Glucocorticoid induced AI may also arise in patients with adrenal malignancies from the use of this treatment as part of the management of the primary neoplastic disease [15].

Due to the commonality of typical symptoms of hypoadrenalism, such as fatigue, anorexia, and nausea, with those of malignant disease, missed AI diagnoses can occur when AI symptoms are misattributed to the effects of the malignancy itself [9, 16]. Rarely, malignancy-associated AI may present as an adrenal crisis (AC), with features including hypotension, electrolyte abnormalities, acute abdominal symptoms, and alterations in consciousness [15, 17]. In these circumstances, urgent treatment with intravenous hydrocortisone and fluid replacement is necessary to relieve symptoms and forestall the development of cardiovascular collapse and possibly death [15].

Despite the relative rarity of AI in patients with an adrenal malignancy, early identification of characteristic AI symptoms, which can be readily treated with glucocorticoid replacement therapy, with or without additional mineralocorticoid, can improve quality of life and avert a potentially fatal AC [3, 7]. However, there is limited information available on the incidence of AI in patients with adrenal malignancies, especially among those with adrenal metastases [4], to guide clinical management of patients. The aim of this study was to investigate patterns of hospitalisation in patients with adrenal malignancies and examine the occurrence of AI/AC in such patients.

## Materials and methods

### Design and data collection

The state of New South Wales (NSW), Australia, has a population of over 7 million people. All documented diseases and procedures for each patient in NSW hospitals (public and private) are coded according to the Australian modification of the International Statistical Classification of Diseases and Related Problems (ICD10 AM) for diagnoses and the Australian Classification of Health Interventions (ACHI) for procedures [18, 19] and are stored by the NSW Ministry of Health in the Admitted Patient Data Collection (APDC). For the purposes of this study, a deidentified unmatched dataset was obtained from the NSW Ministry of Health for the years 2006–2017 for any admission among patients aged 18 years or more to NSW hospitals in which there was either a principal or comorbid diagnosis of a primary or secondary adrenal malignancy. Eligible admissions were identified according to the ICD10 AM rubrics: C79.7 (Secondary malignant neoplasm of adrenal gland), C74.0 (Malignant neoplasm of cortex of adrenal gland), C74.1 (Malignant neoplasm of medulla of adrenal gland), and C74.9 (Malignant neoplasm of adrenal gland, unspecified).

Patient demographic and clinical variables extracted for analysis included: gender; age; admission year; length of hospital stay; all diagnoses and all procedures; whether the patient had an adrenal malignancy or a secondary malignancy in the adrenal with associated primary site; and in-hospital mortality. Comorbid adrenal insufficiency was identified by the ICD 10 AM rubrics: E27.1 (Primary adrenocortical insufficiency), E27.2 (Addisonian crisis), E27.3 (Drug-induced adrenocortical insufficiency), E27.4 (Other and unspecified adrenocortical insufficiency), and an adrenal crisis was located by its rubric: E27.2 (Addisonian crisis). Accompanying signs of AI/AC, where coded, included hyperkalaemia, hyponatraemia, hypotension, and hypoglycaemia. Relevant surgical interventions including adrenalectomy (35600) and nephrectomy (36528) were also identified. There were 161 admissions with a principal diagnosis code of ‘waiting for a suitable diagnosis’ – Z75.1 which were excluded from the study. Admissions for day only investigations or treatments, such as chemotherapy, were not included in this dataset.

Population data, which were used as denominators for calculation of admission and AC event rates, were derived from the HealthStats NSW [20]. Ethics approval, including waiving of the requirement of informed consent, for this deidentified retrospective study of a hospital dataset, was received from the Notre Dame University Human Research Ethics Committee, Reference Number: 018177S. The study was conducted according to the guidelines and regulations of this Committee. As the study involved deidentified data from a state repository consent from individual patients was not required.

### Statistical analysis

Comparisons between demographic variables were conducted using a t test for continuous variables, using appropriate statistics when there was differential variance between the groups, and Chi-square tests for categorical variables. Z-scores were used to compare the rates of admission for primary and secondary adrenal malignancies between the first and last years of the study period. Pearson correlation coefficients were used to assess clinically relevant continuous variables. A 5% level of significance was used. Data analysis was performed using SPSS, V.25 (SPSS Inc., Chicago, Illinois, USA). In addition, for the purposes of analysis, adrenal insufficiency was classified as either primary AI or “other and unspecified AI”.

## Results

### Adrenal malignancy

**Fig. 1:**
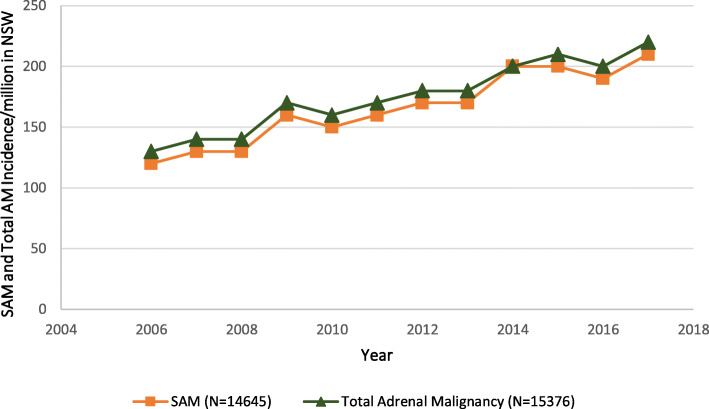
Secondary Adrenal Malignancy (SAM), and Total Adrenal Malignancy (AM) Hospitalisations between 2006 and 2017 in New South Wales (NSW) population, Australia

There were 15,376 admissions to NSW hospitals between 2006 and 2017 by patients with a principal or comorbid diagnosis of either a primary or secondary adrenal malignancy, corresponding to an average of 1281 admissions per year or 175.9 admissions/million population/year. Overall, admissions for any form of adrenal malignancy increased from 129.9/million in 2006 to 215.7/million in 2017 (*p* < 0.01) (Fig. 1). The annual admission rate for secondary adrenal malignancy increased substantially, by 74.8% over the study period, from 119.1/million in 2006 to 208.1/million in 2017 (*p* < 0.01) (Fig. 1).

**Fig. 2:**
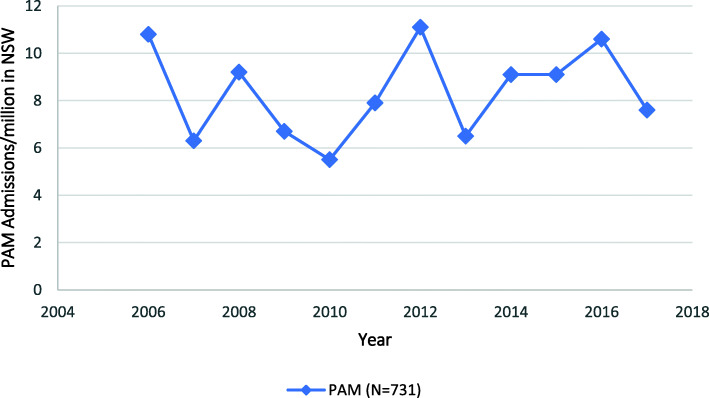
Primary Adrenal Malignancy (PAM), Hospitalisations between 2006 and 2017 in New South Wales (NSW) population, Australia

In contrast, admissions with a diagnosis of primary adrenal malignancy were uncommon and remained steady over the time period, averaging 8.4/million/year (Fig. 2).

**Fig. 3:**
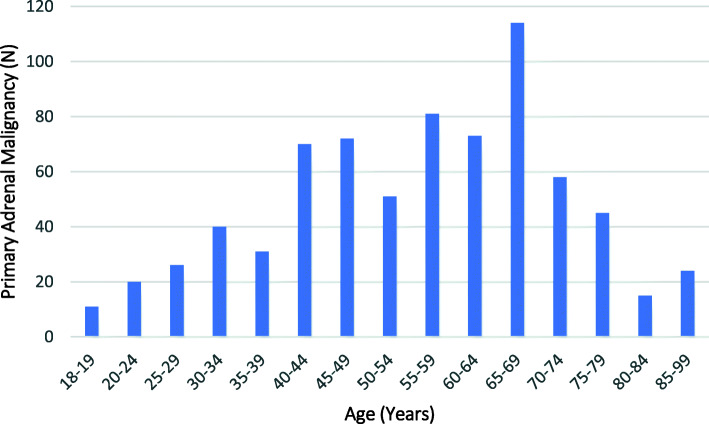
Age Distribution of Patients with a Diagnosis of Primary Adrenal Malignancy (PAM) admitted to NSW Hospitals

**Fig. 4:**
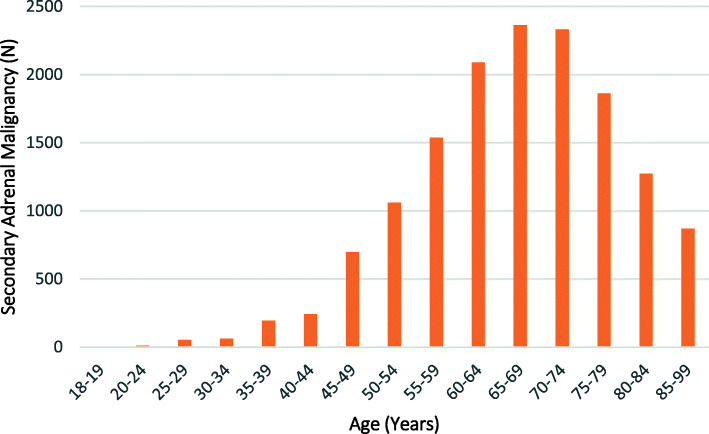
Age Distribution of Patients with a Diagnosis of Secondary Adrenal Malignancies (SAM) admitted to NSW Hospitals

The majority (95.2%, *n* = 14,645) of the admissions were for patients with a principal or comorbid diagnosis of a secondary adrenal malignancy, with only 4.8% (*n* = 731) having a primary adrenal malignancy. Patients with secondary malignancies were significantly older than those with a primary adrenal malignancy; mean age 67.2 (sd = 12.1) compared to 55.8 (sd = 16.6) years, respectively (*p* < 0.01). The majority (63.2%) of patients with a primary adrenal malignancy were aged 40–69 years, while 75.6% of patients with a secondary malignancy were aged between 50 and 79 years (Figs. 3 and 4). Males comprised a greater proportion of admissions for patients with both a primary (55.8%, *n* = 408) or a secondary (62.0%, *n* = 9083) adrenal malignancy.

Among patients with a secondary adrenal malignancy, the most common primary lesion was a malignant neoplasm of the respiratory and intrathoracic organs (*n* = 7450, of which 7430 were malignant neoplasms of bronchus and lung), followed by malignant neoplasm of digestive organs (*n* = 2409) (with the most common being malignant neoplasms of the colon, *n* = 628), melanoma and other malignant neoplasms of skin (*n* = 1392) (with malignant melanoma of the skin being predominant, *n* = 1286), and malignant neoplasm of urinary tract (*n* = 1196) (with the majority being malignant neoplasm of the kidney, *n* = 1035). Less common primary malignancies included: malignant neoplasm of breast (*n* = 688), and malignant neoplasms of male and female genital organs (*n* = 305 and *n* = 211, respectively) (Table 1).

**Table 1:** Primary Malignancy Sites in patients Admitted to Hospital with a Diagnosis of a Secondary Adrenal Malignancy

Primary Site (ICD10 AM codes)	Total N *N* = 15,040	Adrenal Insufficiency *N* = 172*	Adrenal Crisis *N* = 24
	N (%)	N (%)	N (%)
Malignant neoplasms of respiratory and intrathoracic organs (*C30-C39)*	7450 (49.5)	53 (30.8)	4 (16.7)
Malignant neoplasms of digestive organs (*C15-C26)*	2409 (16.0)	20 (11.6)	0
Melanoma and other malignant neoplasms of skin (*C43-C44)*	1392 (9.3)	28 (16.3)	3 (12.5)
Malignant neoplasms of urinary tract (*C64-C68)*	1196 (8.0)	27 (15.7)	2 (8.3)
Malignant neoplasms of ill-defined, secondary and unspecified sites (*C76-C80)*	916 (6.1)	12 (7.0)	8 (33.3)
Malignant neoplasm of breast (C50)	688 (4.6)	18 (10.5)	1 (4.2)
Malignant neoplasms of male genital organs (*C60-C63)*	305 (2.0)	4 (2.3)	3 (12.5)
Malignant neoplasms of female genital organs (*C51-C58)*	211 (1.4)	1 (0.6)	0
Malignant neoplasms of mesothelial and soft tissues (*C45-C49)*	112 (0.7)	0	0
Malignant neoplasms of lip, oral cavity and pharynx (*C00-C14)*	92 (0.6)	1 (0.6)	1 (4.2)
Benign neoplasms (*D10-D36)*	65 (0.4)	3 (1.7)	1 (4.2)
Malignant neoplasms, stated or presumed to be primary, of lymphoid, haematopoietic and related tissue (*C81-C96)*	55 (0.4)	0	0
Malignant neoplasms of thyroid and other endocrine glands (*C73-C75)*	38 (0.3)	1 (0.6)	0
Malignant neoplasms of eye, brain, and other parts of central nervous system (*C69-C72)*	38 (0.3)	0	0
Malignant neoplasms of bone and articular cartilage (*C40-C41)*	34 (0.2)	1 (0.6)	0
Neoplasms of uncertain or unknown behaviour (*D37-D48)*	31 (0.3)	3 (1.7)	1 (4.2)
In situ neoplasms (*D00-D09)*	8 (0.1)	0	0

* Total reflects more than one primary site in some patients

By comparison, among those patients with a metastatic malignancy and comorbid AI, 53 (30.8%) had a primary malignant neoplasm of the respiratory and intrathoracic organs; 28 (16.3%) had melanoma and other malignant neoplasms of skin; 18 (10.5%) had a malignant neoplasm of the breast; and 27 (15.7%) had a malignant neoplasm of the urinary tract (Table 1). Among those patients with melanoma as their primary malignancy and AI, there were significantly more males (89.3%, *n* = 25) than females (11.7%, *n* = 3) (*p* < 0.01). There was no observed increase in AI admissions among patients with melanoma as the primary malignancy pre and post the introduction of immunotherapy for this disorder, although the sample size was small. A male predominance (81.1%, *n* = 42) was also identified in those patients with primary respiratory malignancy and AI (females 18.9%, *n* = 10, *p* < 0.01). Of all patients who had an adrenalectomy recorded, 8 (3.6%) were coded as having AI. In hospital mortality among patients with secondary adrenal malignancies who were admitted with comorbid AI/AC was 16.1% (*n* = 29).

An adrenalectomy was recorded in 220 (1.4%) admissions. Among those patients with a primary adrenal malignancy there were 82 (or 11.2%) who were treated with an adrenalectomy. By comparison, there were 138 adrenalectomies in patients with a diagnosis of a secondary adrenal malignancy, corresponding to only 0.9% of patients in this category. In contrast, a radical nephrectomy was undertaken in 0.3% (*n* = 48) of all patients with an adrenal malignancy. Of these, 16 (33.3%) were in patients with primary adrenal malignancy (corresponding to 2.1% of patients with a primary adrenal malignancy) and 32 (66.7%) were among those with a secondary adrenal malignancy, which corresponded to 0.2% of all patients with metastatic adrenal disease.

While there was no difference between the two malignancy types regarding the duration of hospitalisation, a greater proportion (38.3%, *n* = 5608) of patients with secondary adrenal malignancy than those with primary malignancy (18.7%, *n* = 137) were recorded as receiving palliative care (*p* < 0.01). Death in hospital was recorded in a greater proportion of secondary adrenal malignancy patient admissions (25.2%, *n* = 3686), than primary malignancy admissions (10.8%, *n* = 79) (*p* < 0.01).

### Adrenal insufficiency

**Fig. 5:**
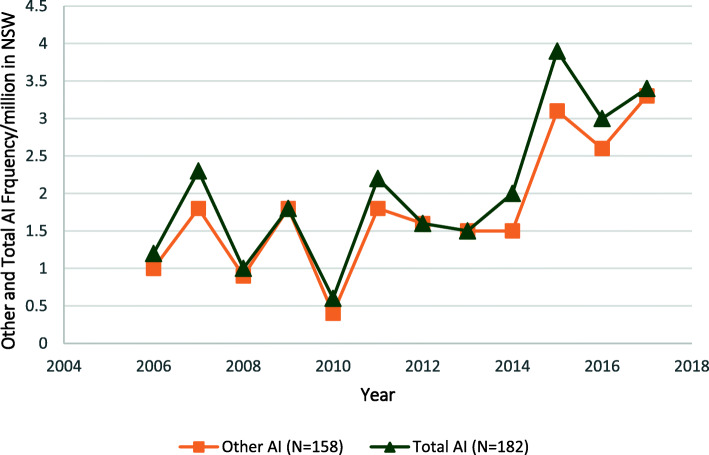
Total Adrenal Insufficiency (AI) Admissions and Admissions for the Category “Other Adrenal Insufficiency” in Association with an Adrenal Malignancy to NSW Hospitals 2006–2017

**Fig. 6:**
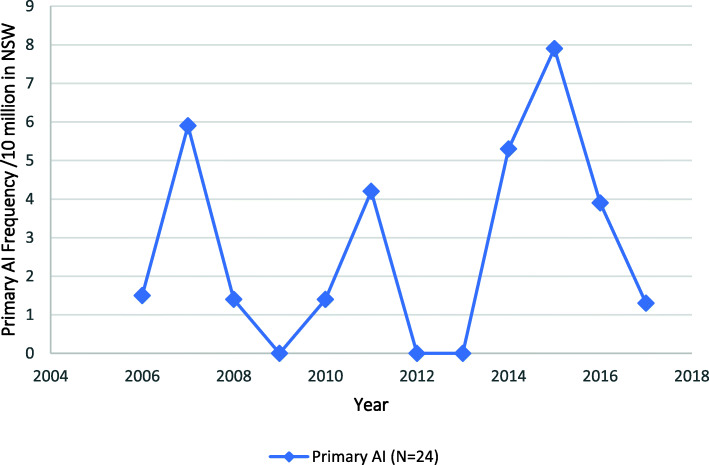
Admissions for the Primary Adrenal Insufficiency in association with an Adrenal Malignancy to NSW Hospitals 2006–2017

Adrenal insufficiency was identified in 182 patients (1.2%) with an adrenal malignancy, corresponding to an annual rate of 15.2 admissions/year, or 2.1/million/year on population basis. The rate of admission for patients with any form of AI increased by 183.3% over the study period, from 1.2/million in 2006 to 3.4/million in 2017 (*p* < 0.01) (Fig. 5). Of those admissions, the majority (86.8%, *n* = 158) were in patients with a diagnosis in the “other and unspecified” category of AI, of whom 8 (4.4%) were for patients with drug-induced AI. Admissions for the “Other and Unspecified” category of AI increased from 1.0/million in 2006 to 3.3/million in 2017 (*p* < 0.01) (Fig. 5). By comparison, a diagnosis of primary AI was found in 24 (13.2%) of all admissions with an AI diagnosis. The yearly admission rates associated with a diagnosis of primary AI did not change and occurred at an average of 0.3/million/year (Fig. 6).

There were 23 admissions in which the patient had a comorbid diagnosis of ‘hypofunction and other disorder of the pituitary gland’, 6 of whom also had a code for comorbid AI. Of the patients with a code for pituitary disease, 12 (52.2%) had hypopituitarism; 10 (43.5%) diabetes insipidus; and there was 1 (4.2%) record which included a code for “disorder of pituitary gland, unspecified”. Despite the increased use of immunotherapy over the time period of the study, there were no records that included an ICD10 AM code for hypophysitis.

Of all patients with a diagnosis of AI in their record, the majority were male (*n* = 121, 66.5%), with a mean age of 65.7 (sd = 12.5) years (Table 2). Symptoms of AI that were recorded among these patients included hyperkalaemia (*n* = 23, 12.6%), hyponatraemia (*n* = 27, 14.8%), hypotension (*n* = 50, 27.5%), and hypoglycaemia (*n* = 7, 3.8%). The majority (79.2, *n* = 19) of the 24 patients with an AC had a secondary adrenal malignancy, with the remaining 5 patients having primary adrenal malignancy (20.8%). Most patients (83.3% *n* = 20) with an AC diagnosis were male and 79.1% (*n* = 19) were aged between 60 and 79 years. Of the symptoms associated with an AC, 37.5% (*n* = 9) were coded as having hypotension, followed by hyperkalaemia (20.8%, *n* = 5), and hyponatremia (16.7%, *n* = 4) (Table 2). Death during that admission was recorded in 4 (16.7%) patients with a diagnosis of AC.

**Table 2:** Adrenal Insufficiency and Adrenal Crisis in Primary and Secondary Adrenal Malignancy In Patients Admitted to Hospital in NSW

	Adrenal Insufficiency *N* = 182	Adrenal Crisis *N* = 24
	N (%)	N (%)
*Adrenal Malignancy*		
Primary	21 (11.5)	5 (20.8)
Secondary	161 (88.5)	19 (79.2)
*Population Frequency /10,000,000/Year*		
Average	20.8	2.7
*Age (years)*		
18–29	1 (1.2)	–
30–39	4 (2.2)	–
40–49	20 (11.0)	2 (8.3)
50–59	32 (17.6)	3 (12.5)
60–69	50 (27.5)	5 (20.8)
70–79	57 (31.3)	14 (58.3)
80+	18 (9.9)	–
Mean (sd)	65.7 (12.5)	68.7 (10.3)
*Gender*		
Male	121 (66.5)	20 (83.3) *
Female	61 (33.5)	4 (16.6)
*Admission Length (days)*		
Median	7	7
Range (IQR)	9	11.25
*In-Patient Mortality*	25 (13.2)	4 (16.7)
*Associated Symptoms*		
Hyperkalaemia	23 (12.6)	5 (20.8)
Hyponatremia	27 (14.8)	4 (16.7)
Hypotension	50 (27.5)	9 (37.5)
Hypoglycaemia	7 (3.8)	–

* *p* < 0.05

## Discussion

This population-based study demonstrated that, over the study period, admission with an adrenal malignancy increased significantly, a phenomenon that was attributable to a substantial increase in admissions among patients with adrenal metastases, while the much smaller number of admissions of patients with a primary adrenal malignancy remained relatively constant. Few (1.2%) of these hospitalisations were associated with a comorbid diagnosis of AI and even fewer (0.2%) included a record of a medically diagnosed AC. In contrast, admissions with comorbid AI for patients with malignant disease of the adrenal gland increased significantly over the duration of the study. Males and older patients were more commonly represented among patients with comorbid AI, and among those patients experiencing an AC. While metastatic adrenal disease was associated with a range of primary neoplasms, those arising from the respiratory tract, melanoma, breast, and urinary tract cancers predominated, and these were associated with the majority of comorbid AI diagnoses.

The data used in this analysis have a number of strengths, including that the dataset comprised a large population-based sample covering a number of years, enabling a sufficient sample size to explore variations in hospitalisations over time. These data do, however, rely on clinical information being entered into the medical record and coded accurately and, while these are subjected to regular quality control checks, it is not possible to be certain that all eligible patients were included in the study dataset. In addition, any patients with undiagnosed AI or those who were not identified as such in the medical record would not be included in the estimates, leading to an underestimate of the true rate of AI in this group. Also, the data did not include information on whether there was unilateral or bilateral involvement of the adrenal glands in any patient with metastatic disease. Not all admissions with AI had the subgroup of AI included in the record, which limited examination of this detail in this population. It was also not possible to determine whether those patients with AI were diagnosed during any one admission or whether they were receiving appropriate therapy. Importantly, these data were not matched for individual patients, and as a result, multiple admissions for the same patient could not be identified, thereby potentially overestimating the occurrence of AI in patients with adrenal malignancies. Despite this, the incidence of AC is a discreet and life-threatening event, that can be documented, and therefore estimated, irrespective of the underlying prevalence of AI, and should be preventable.

The reasons for the observed 74.8% increase in admission rates associated with a metastatic adrenal malignancy, which has not been documented elsewhere, could not be determined in this analysis. However, it is likely that, in the absence of a substantial increase in diagnoses for relevant primary malignancies during this time, the greater use of imaging, both for unrelated symptoms, which was the source of 36.3% of such diagnoses in one recent case series [3]; and staging and follow-up, which characterises the modern management of cancer and was responsible for 58.5% of diagnoses in the same series, [3] may have contributed to an increase in identification of adrenal metastases. By comparison, there were fewer admissions with a primary adrenal malignancy, which showed no clear trend over the same time frame, despite the well documented increase in incidental diagnoses of (mostly benign) adrenal tumours, as a consequence of the increase in imaging for symptoms that are unrelated to adrenal disorders [1, 4].

The results of this analysis suggest that, although higher than the estimated AI prevalence in the general population, diagnosed comorbid AI is relatively rare among hospitalised patients with adrenal malignancies. Males and older age groups were more commonly affected, which is consistent with the epidemiology of the underlying primary malignancies and the generally increased prevalence of AI in the older population [21]. This is in contrast to the proportion of AI patients diagnosed in a recent case series by Mao et al., who identified that, among patients with bilateral metastatic disease, 12.4% of patients had primary AI [3] and to the estimated prevalence of 3–8% in a recent systematic review and meta-analysis [4]. The relative rarity of an AI diagnosis in this study may reflect lower levels of diagnosis or recording of this comorbidity in hospitalised patients. This does not, however, diminish the importance and value of such a diagnosis to an affected patient. Symptoms of AI, including weakness, fatigue, nausea, and vomiting are unpleasant, debilitating and reduce a patient’s quality of life, but can be abolished by implementation of glucocorticoid replacement therapy, which can restore wellbeing rapidly in affected patients. Left untreated, AI can progress to an AC, the cardinal symptom of which is hypotension, that occurs in association with a range of other symptoms including reduced consciousness, acute abdominal symptoms, and electrolyte abnormalities, and is fatal if left untreated [15]. In addition, an AC may be the first presentation of AI in some previously undiagnosed patients with a comorbid malignancy [22]. Although uncommon, there were 24 medically diagnosed and documented AC episodes among the patients in this study. This is probably an underrepresentation of the true AC rate, as there were 41 more admissions in patients with AI in which there was hypotension, suggesting the possibility of an AC in at least some of these cases [15, 17]. Deaths were documented in admissions in which there was a diagnosis of AI/AC, but it is not possible to determine from these data whether this was a relevant factor or whether the death was related to the underlying malignancy.

In this population, an adrenalectomy was conducted on a small proportion of patients but in only 8 patients was this procedure associated with an AI diagnosis. A diagnosis of comorbid hypoadrenalism in this circumstance may be related to a range of factors and, although there have been case reports of symptomatic AI in post-adrenalectomy patients, it is generally considered that unilateral removal of an adrenal gland should not result in the development of clinical AI [23, 24]. Where the adrenalectomy was for a primary adrenocortical cancer, it is likely that at least in some patients, if not all, that management also included the use of the adrenolytic agent, mitotane, which is associated with the development of primary AI [25]. In addition, the use of immune checkpoint inhibitors for some cancers, especially melanoma, some lung cancers and, more recently, renal cell carcinomas has been associated with the development of the endocrinopathies, hypophysitis and less commonly adrenalitis, which are associated with the development of AI. Although the use of these agents is increasing, and hypophysitis is known to occur in under < 1% of patients receiving such treatment, this diagnosis was not identified in this patient sample [15]. In this sample there were also 23 patients with a comorbid diagnosis of ‘hypofunction and other disorders of the pituitary gland’, which might suggest the presence of metastatic pituitary, or non-pituitary intracranial lesions, reinforcing the importance of consideration of pituitary function assessment in suitable patients with metastatic adrenal disease.

There are no clear guidelines on the investigation of possible AI in patients with adrenal metastases, a phenomenon most probably due to the perceived rarity of such a diagnosis that usually occurs at a late stage of metastatic malignancy. Initiation of relevant investigations relies on clinicians considering the possibility of AI in patients with typical symptoms, especially in those cancers that are known to metastasise to the adrenal glands and differentiating these symptoms from those of advanced malignancy [4]. Once diagnosed and treated, all patients with AI should be informed about their AI diagnosis, including the importance of continuation of glucocorticoid therapy and the necessity for medical assistance and parenteral hydrocortisone in circumstances where oral therapy cannot be taken or absorbed, such as when there is vomiting or during the perioperative period [15]. Other strategies that are recommended, including the carriage of a steroid card, possession of injectable hydrocortisone and injection equipment, and use of medical identification jewellery to communicate the AI diagnosis [17], may be relevant to some patients with malignant disease and use of these depends on the clinician and patient’s opinion about the patient’s individual circumstances.

## Conclusions

The results of the present study demonstrated a sustained increase in admissions associated with a diagnosis of metastatic adrenal disease on a population-basis over more than a decade that was accompanied by an increase in AI diagnoses. Adrenal crises, although rare and possibly underdiagnosed, do occur in these patients and are associated with mortality during the admission in which they occurred. Future studies into AI in malignant adrenal disease using matched patient records and the development of cohorts of patients with adrenal malignancies would assist in determining unbiased estimates of AI incidence in these patients. The results of this study serve as a reminder of the possibility of this diagnosis in affected patients and suggest the benefit of the clinician being mindful of an AI diagnosis in a patient with known adrenal metastases and symptoms consistent with AI. Such a diagnosis can be easily managed with glucocorticoid replacement therapy, which can improve quality of life, even in patients with advanced malignant disease.

## Data Availability

The datasets generated and/or analysed during the current study are not publicly available due institutional Ethics Committee requirements but are available from the corresponding author on reasonable request.

## Abbreviations

AM: Adrenal malignancy
AI: Adrenal insufficiency
AC: Adrenal crisis
NSW: New South Wales
ICD10 AM: International Statistical Classification of Diseases and Related Problems
ACHI: Australian Classification of Health Interventions
APDC: Admitted Patient Data Collection

## References

[1] Chandrasekar T, Goldberg H, Klaassen Z, Wallis CJD, Woon DTS, Herrera-Caceres JO, Kulkarni GS, Fleshner NE (2019). The who, when, and why of primary adrenal malignancies: insights into the epidemiology of a rare clinical entity. Cancer.

[2] Iñiguez-Ariza NM (2017). Clinical, biochemical, and radiological characteristics of a single-Center retrospective cohort of 705 large adrenal tumors. *Mayo Clinic proceedings*. Innovations Qual Outcomes.

[3] Mao JJ (2020). Presentation, disease progression and outcomes of adrenal gland metastases. Clin Endocrinol.

[4] Tallis PH, Rushworth RL, Torpy DJ, Falhammar H (2019). Adrenal insufficiency due to bilateral adrenal metastases - a systematic review and meta-analysis. Heliyon.

[5] Ichijo T, Ueshiba H, Nawata H, Yanase T (2020). A nationwide survey of adrenal incidentalomas in Japan: the first report of clinical and epidemiological features. Endocr J.

[6] Mantero F, Terzolo M, Arnaldi G, Osella G, Masini AM, Alì A, Giovagnetti M, Opocher G, Angeli A (2000). A survey on adrenal incidentaloma in Italy. Study group on adrenal tumors of the Italian society of endocrinology. J Clin Endocrinol Metab.

[7] Patrova J, Jarocka I, Wahrenberg H, Falhammar H (2015). Clinical outcomes in adrenal INCIDENTALOMA: experience from one CENTER. Endocr Pract.

[8] Bullock WK, Hirst AE (1953). Metastatic carcinoma of the adrenal. Am J Med Sci.

[9] Kung AW (1990). Addisonian crisis as presenting feature in malignancies. Cancer.

[10] Allolio B, Fassnacht M (2006). Adrenocortical Carcinoma: Clinical Update. J Clin Endocrinol Metab.

[11] Almeida MQ (2018). Primary malignant tumors of the adrenal glands. Clinics (Sao Paulo, Brazil).

[12] Radhakrishnan RK, Mittal BR, Reddy Gorla AK, Malhotra P, Bal A, Varma S (2018). Unilateral primary adrenal lymphoma: uncommon presentation of a rare disease evaluated using (18) F-fluorodeoxyglucose positron emission tomography/computed tomography. World J Nuclear Med.

[13] Salinas C, Renner A, Rojas C, Samtani S, Burotto M (2020). Primary adrenal insufficiency during immune checkpoint inhibitor treatment: case reports and review of the literature. Case Rep Oncol.

[14] Lin CH, Chen KH, Chen KY, Shih SR, Lu JY (2019). Immune checkpoint inhibitor therapy-induced hypophysitis approximately a case series of Taiwanese patients. J Formos Med Assoc.

[15] Rushworth RL, Torpy DJ, Falhammar H (2019). Adrenal crisis. N Engl J Med.

[16] Rosenthal FD, Davies MK, Burden AC (1978). Malignant disease presenting as Addison's disease. Br Med J.

[17] Rushworth RL, Torpy DJ, Falhammar H (2017). Adrenal crises: perspectives and research directions. Endocrine.

[18] National Centre for Classification in Health (2006). International Statistical Classification of Diseases and Related Health Problems, 10th Revision, Australian Modification (ICD-10-AM).

[19] Australian Consortium for Classification Development (ACCD) (2015). The Australian Classification of Health Interventions (ACHI) – Ninth Edition - Tabular list of interventions and Alphabetic index of interventions.

[20] HealthStats NSW (2019). Population by Age, Trends.

[21] Rushworth RL, Torpy DJ, Falhammar H (2020). Adrenal crises in older patients. Lancet Diabetes Endocrinol.

[22] Papierska L, Rabijewski M (2013). Delay in diagnosis of adrenal insufficiency is a frequent cause of adrenal crisis. Int J Endocrinol.

[23] Di Dalmazi G (2014). Adrenal function after adrenalectomy for subclinical hypercortisolism and Cushing's syndrome: a systematic review of the literature. J Clin Endocrinol Metab.

[24] Yoshiji S, Shibue K, Fujii T, Usui T, Hirota K, Taura D, et al. Chronic primary adrenal insufficiency after unilateral adrenonephrectomy: a case report. Medicine. 2017;96(51):–e9091. 10.1097/MD.0000000000009091. 10.1097/MD.0000000000009091 PMC5758139 29390437

[25] Paragliola RM, Torino F, Papi G, Locantore P, Pontecorvi A, Corsello SM (2018). Role of Mitotane in adrenocortical carcinoma - review and state of the art. Eur Endocrinol.

